# Carbidopa-Based Modulation of the Functional Effect of the AAV2-hAADC Gene Therapy in 6-OHDA Lesioned Rats

**DOI:** 10.1371/journal.pone.0122708

**Published:** 2015-04-10

**Authors:** Agnieszka Ciesielska, Nitasha Sharma, Janine Beyer, John Forsayeth, Krystof Bankiewicz

**Affiliations:** Department of Neurological Surgery, University of California San Francisco, San Francisco, California, United State of America; Prince Henry's Institute, AUSTRALIA

## Abstract

Progressively blunted response to L-DOPA in Parkinson’s disease (PD) is a critical factor that complicates long-term pharmacotherapy in view of the central importance of this drug in management of the PD-related motor disturbance. This phenomenon is likely due to progressive loss of one of the key enzymes involved in the biosynthetic pathway for dopamine in the basal ganglia: aromatic L-amino acid decarboxylase (AADC). We have developed a gene therapy based on an adeno-associated virus encoding human AADC (AAV2-hAADC) infused into the Parkinsonian striatum. Although no adverse clinical effects of the AAV2-hAADC gene therapy have been observed so far, the ability to more precisely regulate transgene expression or transgene product activity could be an important long-term safety feature. The present study was designed to define pharmacological regulation of the functional activity of AAV2-hAADC transgene product by manipulating L-DOPA and carbidopa (AADC inhibitor) administration in hemi-parkinsonian rats. Thirty days after unilateral striatal infusion of AAV2-hAADC, animals displayed circling behavior and acceleration of dopamine metabolism in the lesioned striatum after administration of a low dose of L-DOPA (5 mg/kg) co-administered with 1.25 mg/kg of carbidopa. This phenomenon was not observed in control AAV2-GFP-treated rats. Withdrawal of carbidopa from a daily L-DOPA regimen decreased the peripheral L-DOPA pool, resulting in almost total loss of L-DOPA-induced behavioral response in AAV2-hAADC rats and a significant decline in striatal dopamine turnover. The serum L-DOPA level correlated with the magnitude of circling behavior in AAV2-hAADC rats. Additionally, AADC activity in homogenates of lesioned striata transduced by AAV2-AADC was 10-fold higher when compared with AAV2-GFP-treated control striata, confirming functional transduction. Our data suggests that the pharmacological regulation of circulating L-DOPA might be effective in the controlling of function of AAV2-hAADC transgene product in PD gene therapy.

## Introduction

Parkinson’s disease (PD) is a common neurological disorder in which progressive degeneration of dopaminergic neurons in the substantia nigra is responsible for the prominent motor dysfunction seen in the disease [1–3]. Dystrophic failure of dopaminergic neurons is also associated with elimination of the enzymes that catalyze production of dopamine (DA), in particular, aromatic L-amino acid decarboxylase (AADC). Because adequate (non-rate-limiting) levels of AADC are required for efficient conversion of the mainstay drug, L-DOPA (L-3,4-dihydroxyphenylalanine) into DA, progressive failure of L-DOPA therapy becomes apparent in most PD patients within six years after beginning L-DOPA therapy [4, 5], seen in terms of steadily increasing dose accompanied by less predictable response and the appearance of L-DOPA-induced dyskinesias.

A potential solution to this progressive loss of therapeutic effect of L-DOPA is to augment AADC concentrations in the affected striatum (putamen). Accordingly, we have developed over many years a gene therapy that, in preclinical studies in Parkinsonian nonhuman primates, has demonstrated its capacity for driving a dramatic improvement in L-DOPA response [6]. Recombinant adeno-associated virus (AAV2)-based gene therapy offers a way to deliver human AADC transgene with optimal distribution and long-term expression in the affected nigrostriatal pathway [7]. A recent clinical trial indicated that AAV2-hAADC gene therapy is safe [8] and, at the low doses of vector used, evinced encouraging signs of efficacy [9, 10].

Although AAV2-hAADC gene therapy directs clinically relevant restoration of AADC and concomitant reduction in L-DOPA threshold, ongoing nigrostriatal degeneration prevents DA storage. Dopamine in the nigrostriatal system is hence only available, when the blood level of L-DOPA is high enough to drive significant enzymatic conversion. Patients having received AAV2-hAADC are still exposed to pulsatile stimulation of dopamine receptors after oral L-DOPA/carbidopa administration. On that basis, L-DOPA remains the driver of therapeutic benefit in PD patients and thus control of L-DOPA dosing is a key concern.

The present study tested hAADC transgene modulation by means of a simple carbidopa-based regime to regulate therapeutic L-DOPA levels. For functional assessment of the interaction between AAV2-hAADC therapy and L-DOPA/carbidopa, we used the behavioral rotation test in unilaterally 6-hydroxydopamine (6-OHDA)-lesioned rats transduced with AAV2-hAADC vector in order to explore drug dynamics in this setting.

## Material and Methods

### Animals

Male Sprague-Dawley rats (9–10 weeks old) (~250–300 g, Charles River Laboratory) were caged in groups of 3 per cage with 12:12 h light/dark cycle. Temperature and humidity of the animal room was maintained at 19–21°C and 50–60%, respectively. All animals had free access to food and water. All procedures were approved by, and in accordance with, the regulations of the Institutional Animal Care and Use Committee of the University of California, San Francisco (Permit Number: AN091109-03), and all efforts were made to minimize suffering.

### Medial forebrain bundle lesion with 6-hydroxydopamine

All animals (n = 60) were anesthetized with intraperitoneal ketamine (5 mg/kg), and then maintained under 2% isoflurane for the duration of the surgery in a small-animal stereotactic frame (http://www.kopfinstruments.com). The skull was exposed and a burr-hole was created to permit insertion of a cannula for injection of 6-hydroxydopamine (6-OHDA) into the medial forebrain bundle (MFB). Animals received a stereotactically guided injection of 4 μl of 2 mg/ml 6-OHDA in 0.9% saline, infused at a rate of 0.5 μl/min for 10 min into the MFB in the right hemisphere at the following coordinates: antero-posterior (A/P) − 2.2 mm, mediolateral (M/L) 1.5 mm relative to bregma and dura, and ventro-dorsal (V/D) − 8.0 mm [11]. A customized silica cannula (O.D. 235 μm; I.D. 100 μm—Polymicro Technologies, http://www.molex.com) was used for the infusion as previously described [12]. To avoid the backflow of vehicle or 6-OHDA along the needle track, the silica cannula was left in place for additional 2 min after microinjection. The skin incision was closed and post-operative buprenorphine (Buprenex; 0.01 g/kg) was administered subcutaneously as an analgesic. Animals were allowed to recover before returning to the animal housing facilities.

### Rotational testing

Animals treated with 6-OHDA had to meet the following study selection criteria: (i) brisk rotation in response to subcutaneous apomorphine (0.05 mg/kg) and high-dose intra-peritoneal (i.p.) L-3,4-dihydroxyphenylalanine methyl ester hydrochloride (L-DOPA; 10 mg/kg), and (ii) no rotation in response to a sub-therapeutic dose of L-DOPA (5 mg/kg). Four weeks after 6-OHDA intoxication, full 60 min body rotations were recorded and the data are expressed as net full body turns/min, as previously described [12]. Rats exhibiting a rate >5 turns/min in response to 0.05 mg/kg subcutaneous apomorphine (cat. A-4393; www.sigmaaldrich.com) were used for subsequent experimentation (n = 45). Five weeks after 6-OHDA injection, all lesioned animals were tested for L-DOPA/carbidopa response to a sub-therapeutic dose of L-DOPA (cat. D1507; www.sigmaaldrich.com), co-infused i.p. with carbidopa at 1.25 mg/kg (cat. C-1335; www.sigmaaldrich.com) in the same vehicle. The drugs were dissolved in sterile saline (with minimum volume of 0.1 M HCL). Only 3 rats rotated in response to 5 mg/kg L-DOPA/1.25 mg/kg carbidopa and they were excluded from study. Next, we confirmed that the selected lesioned rats responded to a therapeutic dose of L-DOPA (10 mg/kg L-DOPA; 1.25 mg/kg carbidopa). All rats met these criteria and exhibited vigorous rotational responses to this dose.

### AAV2 vectors and vector infusion

Construction of AAV2-hAADC and AAV2-GFP has been described previously [6]. Briefly, both vectors were produced by a triple-transfection technique at the Center for Cellular and Molecular Therapy, Children’s Hospital of Philadelphia Research Vector Core (Philadelphia, PA). AAV2 was infused at a dose of 7 x 10^10^ vector genomes (vg) per injection site.

One week after the exclusion testing (6 weeks post-lesion), rats (n = 20 per vector) received unilateral infusions of AAV2–hAADC or AAV2-GFP (10 μl per hemisphere) into the right striatum (ST) at the following stereotactic coordinates relative to the bregma and dura: (AP +0.8, ML 3.0, DV -5.0) [11]. Vector infusions were performed with the same intracerebral infusion system as described above for the 6-OHDA lesions (flow rate: 0.5 μl/min for 20 min).

### Drug administration and behavioral testing

The influence of carbidopa regimen on L-DOPA-induced rotational behavior and biochemical consequences of carbidopa withdrawal were studied in all transduced rats. Four weeks after AAV2-GFP or AAV2-hAADC injection, daily sub-therapeutic injections of L-DOPA (5 mg/kg plus carbidopa 1.25 mg/kg i.p.) were initiated and continued for 10 days. On Day 8, rats were injected with only L-DOPA (no carbidopa). Rotational behavior was recorded for 120 min on days 1, 5, 7, 8 and 9. Total rotational activity was measured by counting net counterclockwise rotations.

### Blood sampling

After completion of the final behavioral test, animals were anaesthetized with isoflurane and basal blood was obtained from the tail vein. Immediately after basal blood collection, 5 mg/kg L-DOPA plus 1.25 mg/kg carbidopa or 5 mg/kg L-DOPA alone were injected i.p. into AAV2-hAADC or AAV2-GFP transduced rats (n = 5 per group). Blood samples were collected by tail venipuncture 0, 5, 15, 30, and 60 min after drug injection. Blood was collected into serum separation tubes and was centrifuged 3000 x g for 10 minutes. Rats were sacrificed by decapitation after the last blood collection, i.e. 60 min after drug administration. The dissected left and right striata (for monoamines concentration) alongside serum samples (~200 μl) were stored at -80°C for subsequent high performance liquid chromatography (HPLC). For AADC activity assay left and right striata were dissected from AAV2-GFP or AAV2-hAADC rats (n = 4 per group) 1 hour after injection of 5 mg/kg L-DOPA plus 1.25 mg/kg carbidopa or 5 mg/kg L-DOPA alone (without blood samples collection). Additionally, 2 rats from each vehicle group were transcardially perfused and the brains were processed for immunohistochemistry [13].

### Determination of serum and striatal levels of monoamines and striatal AADC activity

For determination of monoamines level serum samples were prepared as described in [14]. Briefly, 2 μl of 7 M perchloric acid (PCA) was mixed with 150 μl of serum. Striatal samples were homogenized by sonication in 0.4 M PCA. Serum or striatal samples were centrifuged at 14,000 x g for 15 min at 4°C and the supernatants were filtered (0.22 μm pore size filter, cat. UFC30LG25; www.emdmillipore.com) and kept at -80°C for HPLC analysis.

*In vitro* AADC activity assay is based on the measurement of the enzymatic formation of DA from L-DOPA via an HPLC-based assay [15, 16]. Striatal tissue was homogenized in 10 volumes of 0.25 M sucrose by mechanical disruption with glass beads (0.1 mm) in a Bullet Blender (http://www.nextadvance.com). The homogenates were centrifuged at 6000 x g for 20 min at 4°C. After a second centrifugation (10.000 x g for 10 min at 4°C), the supernatant was collected and held on wet ice. To measure enzymatic conversion of L-DOPA into dopamine, 40 μl of the supernatant was added to 360 μl of an incubation mixture containing 50 mM sodium phosphate buffer, pH 7.2; 0.1 mM ascorbic acid, 0.1 mM pyridoxal-5’-phosphate, 1 mM DL-dithiothreitol, 0.1 mM EDTA, and 0.1 mM pargyline. All reagents were purchased from Sigma-Aldrich (www.sigmaaldrich.com). Samples were pre-incubated at 37°C for 5 min and the reaction was initiated by addition of 20 μl of L-DOPA (0.3 mM final concentration) and allowed to proceed for 30 min at 37°C, whereupon it was stopped by addition of 80 μl ice-cold 0.4 M PCA. The mixture was then centrifuged at 3000 x g for 10 min. The supernatant was stored at -80°C until further HPLC analysis. Non-enzymatic conversion was determined by substituting water for the L-DOPA substrate (blank samples). Some homogenates were spiked with L-DOPA solution and benserazide (0.01 mM) to serve as a control.

Serum and striatal samples were applied to HPLC coupled to an electrochemical detector (ECD) (ECD, CoulArray 5600A, cat. 70–4320, www.dionex.com) for determination of monoamines: L-DOPA, DA, 3,4-dihydroxyphenylacetic acid (DOPAC), homovanillic acid (HVA), 5-hydroxytryptamine (5-HT) and 5-hydroxyindoleacetic acid (5-HIAA). All standards were purchased in Sigma-Aldrich (www.sigmaaldrich.com—L-DOPA: cat. D-9628; DA: cat. H8502; DOPAC: cat. 850217; HVA: cat. H1252; 5-HT: cat. H9523; 5-HIAA: cat. H8876) Supernatant (30 μl) was injected onto the HPLC-ECD system and was separated on a reverse-phase, analytical column MD-150X3.2 (cat. 70–0636; www.dionex.com) with a mobile MD-TM phase (cat. 70–1332; www.dionex.com) and flow rate of 0.5 ml/min. In parallel with serum and striatal samples, a series of monoamine standard solutions with known concentration (1000 ng/ml, 100 ng/ml and 20 ng/ml) were also chromatographed. Monoamine in the samples were identified by retention times of standards and quantified by measuring the area under the peaks using software Coularray Data Station 3.00 (www.dionex.com). Concentrations of monoamine in examined samples were reported by comparison with a monoamine standards curve and were expressed either as ng/ml in serum samples or as ng per mg protein in striatal samples. The protein concentrations of tissue homogenates were measured accordingly to the Bio-Rad DC protein analysis protocol (http://www.bio-rad.com/LifeScience/pdf/Bulletin_9005.pdf) and Perkin Elmer Bio Assay Reader.

### Immunohistochemistry

Fixed brains were cut into serial 40-μm coronal sections with a freezing microtome. Sections were collected in sequence (20 sets of sections), stored in 24-well plates in cryoprotectant solution (0.5 M sodium phosphate buffer, pH 7.4, 30% glycerol and 30% ethylene glycol) at 4°C until further processing. Immunohistochemistry was performed on free-floating sections. Briefly, washes with PBS were performed between each immunohistochemical step. Endogenous peroxidase activity was quenched for 30 minutes in 1% v/v hydrogen peroxide in PBS. The blocking of unspecific binding was achieved with Background Sniper (cat. BS966MM; www.biocare.net) for 30 minutes at room temperature (RT). Thereafter, sections were incubated overnight with specific primary antibodies: polyclonal rabbit anti-human AADC, 1:5000 (cat. AB136; www.emdmillipore.com); monoclonal rabbit anti-GFP, 1:500 (cat. AB3080; www.emdmillipore.com). Antibodies were dissolved in DaVinci diluent (cat. PP900M; www.biocare.net). Sections were incubated with Mach 2 anti-rabbit horseradish peroxidase (HRP) polymer (cat. RHRP520L; www.biocare.net) for 1 h at RT. HRP activity was visualized with commercially available kit with 3,3′-diaminobenzidine (DAB) peroxide substrate (cat. SK4100; www.vectorlabs.com). Finally, immunostained sections were mounted on gelatinized slides, dehydrated in alcohol and xylene and cover-slipped with Cytoseal (cat. 23–244257; www.fishersci.com). All sections were examined and digitally photographed on a Zeiss Axioskop microscope.

### Data analysis

Analyses are expressed as mean ± S.E.M. Behavioral data were analyzed with non-parametric Kruskal Wallis analysis of variance followed by Mann-Whitney post-hoc test. Effects of treatments on monoamine levels and striatal AADC activity were analyzed by ANOVA followed by multiple comparisons with the Fisher’s LSD post-hoc test. The correlation between the serum concentration of L-DOPA and L-DOPA-induced behavior during the first 60 minutes in AAV2-hAADC rats was examined by Pearson correlation. Differences were considered to be significant when p<0.05. Each test was calculated with STATISTICA software (www.statsoft.pl).

## Results

### Regulation of L-DOPA-induced response in AAV2-hAADC rats by carbidopa

**Fig 1:**
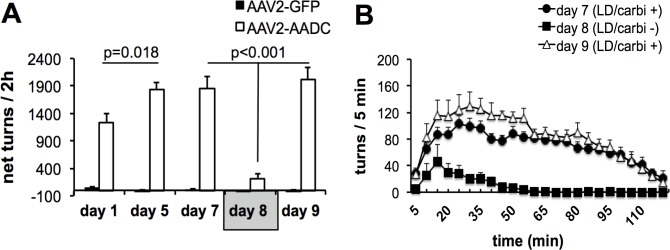
Effect of acute withdrawal of carbidopa (1.25 mg/kg) from daily L-DOPA regimen (5 mg/kg), on L-DOPA induced rotational response in AAV2-hAADC and AAV2-GFP rats. (A) Total number of contralateral rotations exhibited in 2 h, expressed as net contralateral rotations in AAV2-hAADC rats and AAV2-GFP rats on treatment days 1, 5, 7, 8 (acute carbidopa withdrawal day) and 9, respectively. (B) Time-course of rotational responses on day 7, 8 and 9 of L-DOPA administration with or without carbidopa. Data are represented as mean ± S.E.M. Significant differences were determined by non-parametric Kruskal Wallis analysis of variance at each test day with Mann-Whitney post-hoc.

Before acute carbidopa withdrawal, all rats transduced with AAV2-GFP or AAV2-hAADC were injected daily with 5 mg/kg L-DOPA in combination with 1.25 mg/kg carbidopa for 7 days. In AAV2-GFP rats, no significant behavioral effects were observed over the entire period of L-DOPA/carbidopa administration (Fig 1A). In the AAV2-hAADC group, robust contralateral rotation was evoked in all animals on the first day of L-DOPA/carbidopa treatment (Fig 1A). As expected with daily L-DOPA/carbidopa administration, the rotational response to L-DOPA/carbidopa increased progressively during the first few days of treatment and stabilized from day 5 after drug injection onwards (Fig 1A). Therefore, the effect of acute carbidopa withdrawal was studied during the stable phase of L-DOPA/carbidopa circling response in AAV2-hAADC rats.

The absence of carbidopa in the L-DOPA regimen (day 8) prevented the induction of contralateral turning in 9 of 12 AAV2-hAADC animals (net turns ± SEM: 39 ± 13.2 per 120 min). In absence of carbidopa, L-DOPA-induced turning behavior was still observed in three animals (included in analysis). However, the total number of turns was approximately 53% less than that in the L-DOPA/carbidopa testing session. Acute withdrawal of carbidopa resulted in an approximate 87% reduction in L-DOPA-induced contralateral turning in AAV2-hAADC rats (Fig 1A and 1B). Reintroduction of carbidopa to L-DOPA treatment regimen (day 9) restored pronounced contralateral rotations in AAV2-hAADC rats (Fig 1A and 1B). Additional preliminary data showed the behavioral effect of acute carbidopa withdrawal is stable and repeatable (S1 Fig).

### Effect of carbidopa withdrawal on L-DOPA serum level

**Fig 2:**
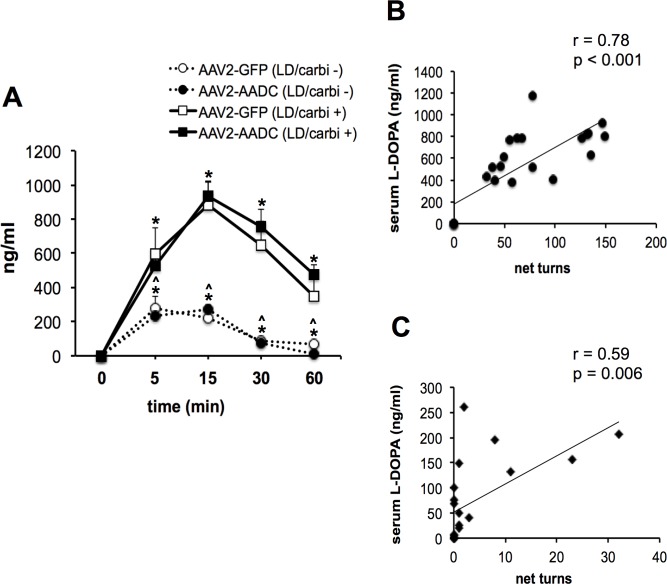
Serum L-DOPA levels. (A) Time-course of serum L-DOPA levels after last L-DOPA administration at dose 5 mg/kg (day 10) with co-injection of 1.25 mg/kg carbidopa (n = 5 per AAV2-hAADC or AAV2-GFP group) and without carbidopa co-injection (n = 5 per AAV2-hAADC or AAV2-GFP group). Data are presented as mean ± S.E.M. Significant differences were determined by Fisher’s LSD post-hoc test. * p<0.01 compared with baseline (time 0); ^#^ p<0.01 compared with rats treated with L-DOPA plus carbidopa. Serum L-DOPA level was significantly correlated with L-DOPA-induced rotation in the initial 60 minutes after administration of (B) L-DOPA/carbidopa or (C) L-DOPA alone.

Fig 2 shows the time-course of the serum L-DOPA levels after repeated administration of L-DOPA with carbidopa (5/1.25 mg/kg) or L-DOPA alone in AAV2-GFP or AAV2-hAADC rats. In both AAV2-hAADC and AAV2-GFP rats, the peak concentrations were observed at 15 minutes after L-DOPA/carbidopa injection and between 5–15 minutes after those administered only L-DOPA. The peak concentration was significantly higher in L-DOPA/carbidopa treated groups than L-DOPA-induced rats without carbidopa administration (about 85%). In both groups of rats, treatment with L-DOPA alone resulted in rapid reduction of serum L-DOPA concentration to the baseline level at 60 minutes post-injection (Fig 2). Additionally, we observed that serum L-DOPA levels correlated with L-DOPA-induced circling response in the first 60 minutes in AAV2-hAADC rats (Fig 2B and 2C).

### Effect of carbidopa withdrawal on striatal monoamine concentration and turnover

The concentration of monoamines detected in lesioned striata 1 hour after systemic administration of 5 mg/kg L-DOPA with 1.25 mg/kg carbidopa or L-DOPA alone, are summarized in Table 1. The striatal L-DOPA concentration was significantly lower in AAV2-hAADC rats treated with L-DOPA/carbidopa, compared with control AAV2-GFP rats. This might indicate increased consumption of L-DOPA in lesioned striatum transduced with AAV2-hAADC. Interestingly, no significant influence of carbidopa withdrawal was detected on striatal L-DOPA concentration in any group. Striatal DA concentration remained at an equally low level after L-DOPA/carbidopa treatment in both the AAV2-hAADC and control AAV2-GFP groups. Within the same groups, withdrawal of carbidopa resulted in a significant reduction of DA levels. DOPAC and HVA were significantly increased in AAV2-hAADC rats treated with L-DOPA/carbidopa compared with AAV2-GFP animals treated in the same manner. The absence of carbidopa in the L-DOPA challenge reduced both DOPAC and HVA in lesioned striata in both AAV2-hAADC and AAV2-GFP rats. The DOPAC/DA, HVA/DA and DOPAC+HVA/DA ratios indicated a substantial increase in striatal dopamine turnover in AAV2-hAADC rats treated with L-DOPA/carbidopa compared with AAV2-GFP animals (Table 2). The enhanced DA metabolism in lesioned striatum was significantly reduced after withdrawal of carbidopa in both AAV2-hAADC and AAV2-GFP rats; however, this effect was more pronounced in AAV2-hAADC rats (HVA/DA: 53% reduction in AAV2-hAADC *vs* 29% in AAV2-GFP, *p<0*.*01* and DOPAC+HVA/DA ratio: 54% reduction in AAV2-hAADC vs 36% in AAV2-GFP, *p<0*.*05*).

**Table 1:** Striatal concentrations of monoamines, 1 hour after injection of 5 mg/kg L-DOPA with or without 1.25 mg/kg carbidopa in hemi-parkinsonian rat after unilateral transduction with AAV2-GFP or AAV2-hAADC vector.

Group	Treatment	Monoamines (ng/mg protein)					
		*L-DOPA*	*DA*	*DOPAC*	*HVA*	*5-HT*	*5-HIAA*
*Lesioned striatum*							
AAV2-GFP	LD/carbi +	1.15 ± 0.30	1.89 ± 0.07	0.97 ± 0.16	1.71 ± 0.06	6.22 ± 0.80	6.82 ± 0.5
	LD/carbi -	1.22 ± 1.30	0.97 ± 0.12 ^b^	0.27 ± 0.03 ^b^	0.67 ± 0.18 ^b^	7.92 ± 0.60	6.51 ± 0.6
AAV2-hAADC	LD/carbi +	0.32 ± 0.12 ^a^	1.87 ± 0.12	1.39 ± 0.15 ^a^	2.25 ± 0.05 ^a^	5.96 ± 0.53	6.52 ± 0.8
	LD/carbi -	0.17 ± 0.15	1.11 ± 0.12 ^b^	0.36 ± 0.04 ^b^	0.65 ± 0.14 ^b^	7.89 ± 1.00	6.03 ± 0.4
*Un-lesioned striatum*							
AAV2-GFP	LD/carbi +	0.45 ± 0.05	153.9 ± 5.70	15.5 ± 0.60	11.3 ± 0.40	3.12 ± 0.30	3.68 ± 0.2
	LD/carbi -	0.57 ± 0.17	111.5 ± 11.5 ^b^	12.4 ± 2.80	7.1 ± 1.10 ^b^	3.43 ± 0.40	3.51 ± 0.5
AAV2-hAADC	LD/carbi +	0.61 ± 0.08	148.8 ± 11.1	12.7 ± 1.32	9.6 ± 0.71	3.86 ± 0.51	4.15 ± 0.4
	LD/carbi -	n.d.	132.3 ± 10.6	10.9 ± 1.56	7.3 ± 0.89 ^b^	3.10 ± 0.32	3.49 ± 0.3

Data are expressed as mean and S.E.M. (n = 5 per group)

Confidence intervals are indicated as follows:

^a^ p<0.05 compared to AAV2-GFP treated with L-DOPA/carbidopa + (LD/carbi +) group

^b^ p<0.01 compared to LD/carbi + groups

**Table 2:** Metabolic rate of striatal monoamines, 1 hour after injection of 5 mg/kg L-DOPA with or without 1.25 mg/kg carbidopa in hemi-parkinsonian rat after unilateral transduction with AAV2-GFP or AAV2-hAADC vector.

Group	Treatment	Metabolic rate of monoamines			
		*DOPAC / DA*	*HVA / DA*	*DOPAC+HVA / DA*	*5-HIAA / 5-HT*
*Lesioned striatum*					
AAV2-GFP	LD/carbi +	0.52 ± 0.08	0.81 ± 0.04	1.33 ± 0.10	1.05 ± 0.10
	LD/carbi -	0.27 ± 0.01 ^b^	0.65 ± 0.13 ^b^	0.92 ± 0.13 ^b^	0.82 ± 0.05 ^b^
AAV2-hAADC	LD/carbi +	0.74 ± 0.02 ^a^	1.27 ± 0.09 ^a^	1.95 ± 0.10 ^a^	1.12 ± 0.05
	LD/carbi -	0.32 ± 0.06 ^b^	0.61 ± 0.12 ^b^	0.92 ± 0.15 ^b^	0.94 ± 0.09 ^b^
*Un-lesioned striatum*					
AAV2-GFP	LD/carbi +	0.10 ± 0.01	0.07 ± 0.01	0.17 ± 0.01	0.98 ± 0.56
	LD/carbi -	0.11 ± 0.01	0.07 ± 0.02	0.17 ± 0.03	0.94 ± 1.15
AAV2-hAADC	LD/carbi +	0.08 ± 0.01	0.06 ± 0.01	0.14 ± 0.02	1.07 ± 0.06
	LD/carbi -	0.08 ± 0.01	0.05 ± 0.01	0.13 ± 0.01	1.06 ± 0.08

Data are expressed as mean and S.E.M. (n = 5 per group)

Confidence intervals are indicated as follows:

^a^ p<0.05 compared to AAV2-GFP treated with L-DOPA/carbidopa + (LD/carbi +) group

^b^ p<0.01 compared to LD/carbi + groups

There was no significant difference in 5-HT and its metabolite, 5-HIAA, between AAV2-hAADC and AAV2-GFP rats, both treated with L-DOPA/carbidopa (Table 1). However, the withdrawal of carbidopa from the L-DOPA regimen, in both AAV2-hAADC and AAV2-GFP rats, reduced the 5-HIAA/5-HT ratio compared with animals injected with L-DOPA/carbidopa (Table 2). The level of this reduction was similar between AAV2-hAADC and AAV2-GFP rats.

We also assessed monoamine levels and turnover in the contralateral control (no vector) striata. In the intact striata of AAV2-hAADC or AAV2-GFP rats, we were able to detect low levels of L-DOPA, regardless of drug regimen, i.e. L-DOPA/carbidopa or L-DOPA alone. Absence of carbidopa from L-DOPA treatment led to a slight reduction in DA and HVA levels in both AAV2-hAADC and AAV2-GFP rats. There were no statistically reliable differences in dopamine and serotonin metabolism between AAV2-hAADC and AAV2-GFP rats treated with L-DOPA/carbidopa or L-DOPA alone (Table 2).

### Effect of AAV2-hAADC transduction on striatal AADC activity

*In-vitro* AADC activity measured in striatal tissue extracts are summarized in Table 3. As expected [17, 18], almost complete dopaminergic neurodegeneration was produced by injecting 6-OHDA into MFB, reduces striatal AADC activity in AAV2-GFP control rats by approximately 80% (compared with those in the contralateral non-lesioned hemisphere). In contrast, HPLC analysis revealed robust formation of DA from exogenous L-DOPA in homogenates of striata from AAV2-hAADC transduced rats. AADC activity in AAV2-hAADC lesioned striatum was elevated several-fold compared to AAV2-GFP lesioned striatum and also with the intact striatum, confirming the effective AAV2-hAADC transduction of the 6-OHDA lesioned striatum.

**Table 3:** AADC activity in the striatum with L-DOPA as substrate, 1 hour after injection of 5 mg/kg L-DOPA with or without 1.25 mg/kg carbidopa in hemi-parkinsonian rat after unilateral transduction with AAV2-GFP or AAV2-hAADC vector.

Group	Treatment	AADC activity (pmol/min/mg protein)	
		*Lesioned striatum*	*Non-lesioned striatum(no vector)*
AAV2-GFP	LD/carbi +	66.44 ± 19.44	370.98 ± 16.87 ^c^
	LD/carbi -	93.82 ± 17.37	361.92 ± 23.69 ^c^
AAV2-AADC	LD/carbi +	877.47 ± 0.12 ^a^	399.13 ± 28.29 ^c^
	LD/carbi -	780.09 ± 204.36 ^b^	389.87 ± 15.71 ^c^

Data are expressed as mean and S.E.M. (n = 4 per group)

Confidence intervals are indicated as follows:

^a^ p<0.05 compared to AAV2-GFP treated with L-DOPA/carbidopa + (LD/carbi +) group

^b^ p<0.01 compared to AAV2-GFP treated with L-DOPA/carbidopa—(LD/carbi-) group

^c^ p<0.01 compared to lesioned striatum

Intra-peritonal administration of 1.25 mg/kg carbidopa with L-DOPA did not alter striatal AADC activity in either AAV2-GFP groups or AAV2-hAADC groups (Table 3). In accord with published results [16], this dose 1.25 mg/kg carbidopa injected i.p, is too low to affect central AADC activity.

### Relative transduction efficiency of AAV2-hAADC

**Fig 3:**
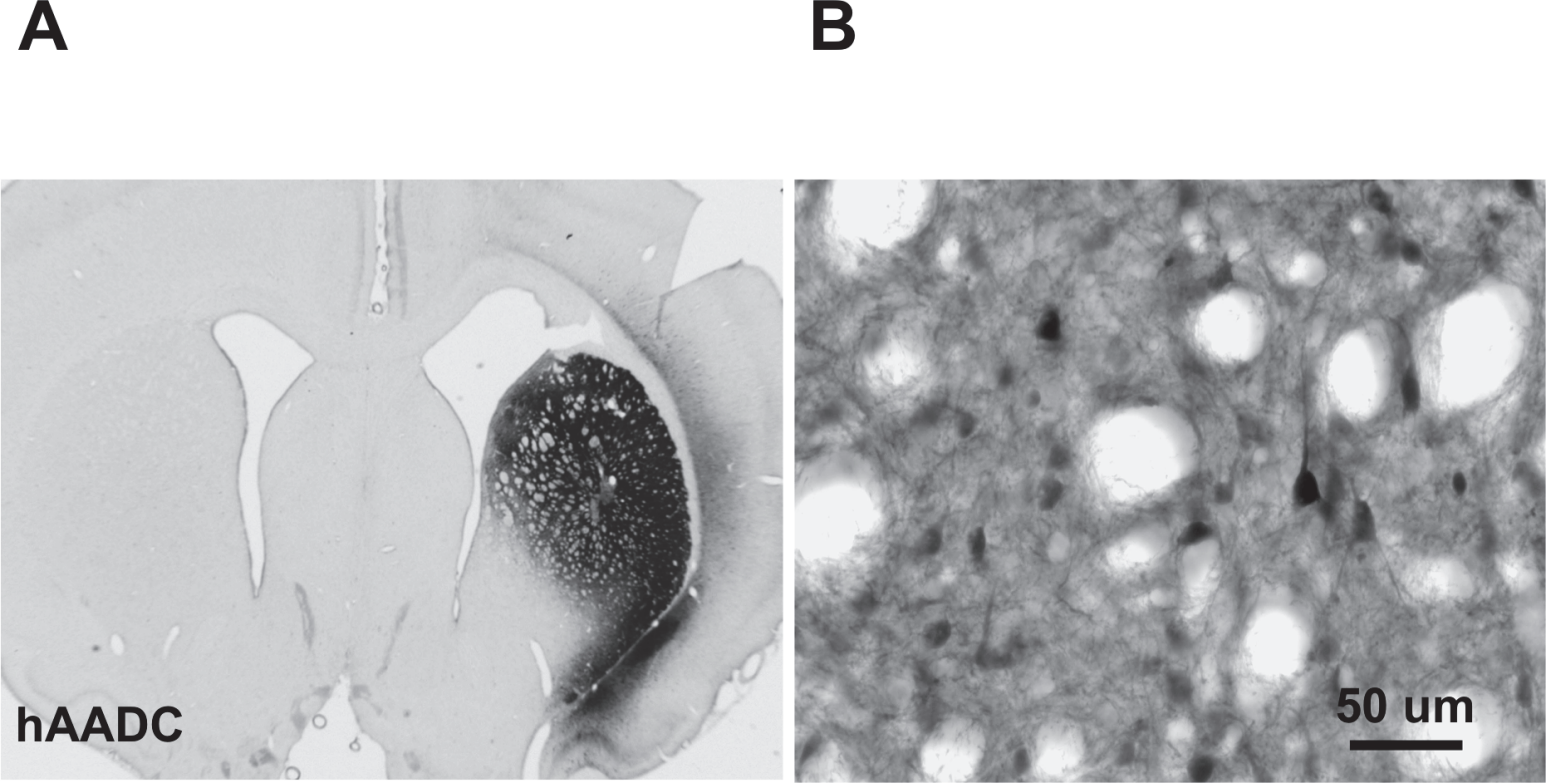
The efficacy of striatal transduction post AAV2-hAADC injection. (A) Human AADC expression was detected almost in entire striatum. (B) Higher magnification reveals individual transduced cells with neuronal morphology. Scale bar = 1000 μm for A; and 50 μm for B.

In some animals, we assessed the efficiency of transduction by AAV2-hAADC by immunohistochemistry. Five weeks after transduction, AAV2-hAADC vector produced intense and widespread hAADC expression throughout the entire striatum (Fig 3). As previously reported [13], hAADC immuno-positive cells displayed a neuronal morphology (Fig 3B).

## Discussion

The mainstay treatment for Parkinson’s disease is L-DOPA. Initially, this precursor of dopamine is very effective in limiting the cardinal symptoms of the disease, but its effectiveness wears off over a number of years as the disease worsens. The enzyme AADC is responsible for the conversion of L-DOPA to dopamine, its active metabolite. As PD progresses, increasing loss of nigrostriatal neurons leads to a progressive inability to convert L-DOPA to dopamine. This resistance to L-DOPA, it has been suggested, results in over-stimulation of the relatively spared limbic system with even higher doses of L-DOPA with a correspondingly blunted effect in the more severely degenerated putamen. It was, therefore, hoped to use gene therapy to restore normal levels of AADC in the striatum of PD patients, and thereby obtain an improved response to levodopa, reduced response fluctuations, and reduced levodopa-induced dyskinesias [7]. Basic enzymology predicts that even rate-limiting amounts of AADC will provide some residual capacity to catalyze dopamine production. However, the use of AAV2-hAADC to direct production of a non-rate-limiting concentration of AADC in the putamen should restore a fully normalized L-DOPA response, obviating the need for excessive L-DOPA dosing [6].

A phase 1 clinical trial of AAV2-hAADC was recently completed. The study involved 10 patients with moderately advanced PD who received either a low dose or a high dose of AAV2-hAADC infused bilaterally into the putamen [8, 9]. A unique advantage of this therapy is that a specific PET ligand, [^18^F]-fluoro-meta-tyrosine (FMT), can be used to assess AADC expression in the brains of study subjects, since FMT is a specific substrate for AADC [6, 19–22]. Thus, subjects were scanned pre-operatively and at intervals after surgery. In all cases, dose-dependent increases in PET signal were seen, and subjects have all shown some improvement in clinical response to levodopa [10]. The therapy appeared to improve mean scores on standardized rating scales by approximately 30% in the on and off state. Interestingly, the two subjects with the greatest increase in PET signal also showed the greatest clinical improvement.

Here we show, in agreement with previous results [22, 23] that delivery of AAV2-hAADC into the lesioned striatum of the rat leads to restoration of behavioral responses to exogenous L-DOPA at sub-therapeutic doses. In our study we used the standard combination of L-DOPA and carbidopa in a 4:1 ratio, which gives the best therapeutic profile [24]. Elimination of carbidopa from a daily L-DOPA/carbidopa regimen results in almost completely suppression of L-DOPA-induced rotation in AAV2-hAADC rats. In agreement with published data [25], the elimination of carbidopa from a daily L-DOPA regimen leads to a reduction in the serum level of L-DOPA below the therapeutic range in AAV2-hAADC rats. One hour after L-DOPA injection alone, serum L-DOPA level reached only 5% of the L-DOPA level observed with L-DOPA/carbidopa administration. Moreover, our study demonstrated the correlation between the blood level of L-DOPA and the rate of turning in AAV2-hAADC rats (increase in rotational counts is an indicator of functional activity of the hAADC transgene related to prominent increase of DA level in lesioned nigrostriatal pathway). These results showed that carbidopa treatment maintains an efficacious serum level of L-DOPA, which seems to be crucial to yield effective L-DOPA concentration in the lesioned striatum and induce contralateral rotation in AAV2-hAADC rats. Moreover, the threshold of this effective serum level of L-DOPA in AAV2-hAADC rats is decreased compared with AAV2-GFP rats.

Although some researchers have reported a poor correlation between plasma level of L-DOPA and clinical improvement ratings in PD and the development of the fluctuating L-DOPA response [26–28], others have reported a strong relationship between the time-dependent peripheral availability of L-DOPA and improvement in motor function [29–31]. The relationship between the pharmacokinetics of L-DOPA and therapeutic response is especially visible at more advanced stages of PD, where modest changes in plasma concentration of L-DOPA can significantly affect the therapeutic response [32]. These results predict that management of circulating L-DOPA concentration (optimally a sustained blood level of L-DOPA) may be critical in controlling L-DOPA efficacy and side effects associated with long-term L-DOPA use. From the perspective of AAV2-hAADC gene therapy in PD, the ability to regulate the bioavailability of L-DOPA will likely be an important factor in optimizing the therapeutic efficacy of this gene therapy. Carbidopa-dependent increases in the peripheral pool of L-DOPA for active transport across the blood brain barrier caused expected DA changes in the dopaminergic system. Post-mortem assessment of striatal monoamines indicates that AAV2-hAADC transduction leads to the marked acceleration of DA catabolism in lesioned striatum after repeated L-DOPA/carbidopa administration, manifested by increases in the level and turnover of major DA metabolites: DOPAC and HVA. This acceleration of metabolic turnover of DA in lesioned striatum transduced with AAV2-hAADC was assumed to be due to increased conversion of L-DOPA to DA. As expected, our *in vitro* study shows that AAV2-hAADC transduction caused efficient increase of AADC activity in lesioned striatum. However, our analysis did not reveal any detectable differences between tissue DA levels in AAV2-hAADC versus AAV2-GFP rats, as reported previously [23]. Thus, AAV2-hAADC transduction does not improve retention of DA in the lesioned striatum. On the other hand, the lack of changes in endogenous DA level, might be partly explained by the use of post-mortem tissue for analysis. In contrast, *in-vivo* microdialysis allows cumulative measures of short-lived extracellular dopamine over a substantial time period, e.g. 15–30 min, but the postmortem method measurement occurs at a single time-point and includes intracellular dopamine [33]. Because post-mortem analysis of monoamines was performed after the peak absorption period of L-DOPA in serum, we assumed that this phenomenon could be responsible for relatively low levels of striatal L-DOPA observed in our HPLC analysis. Additionally, L-DOPA was significantly diminished in lesioned striatum transduced with AAV2-hAADC and this may be related to the increased consumption of L-DOPA by active transgene product.

Withdrawal of carbidopa from L-DOPA regimen resulted in significant reduction of DA and its metabolites in lesioned striatum transduced with AAV2-hAADC. One hour after injection of L-DOPA alone, the level of striatal dopaminergic activity in AAV2-hAADC rats was similar to that observed in AAV2-GFP rats. Additionally, for the first time, we demonstrated that AAV2-hAADC gene therapy in lesioned rats does not directly affect the striatal serotonergic system.

In conclusion, our results suggest that effectiveness of L-DOPA in AAV2-hAADC gene therapy of PD is dependent on the presence of carbidopa. Thus, carbidopa can play a role as an "on-switch" for AAV2-AADC-related therapeutic machinery in lesioned nigrostriatal system. In the near future, we plan to develop a continuous L-DOPA delivery methodology in AAV2-hAADC-treated patients to achieve steady plasma and brain levels of L-DOPA that, when combined with peripheral administration of carbidopa, would allow us to regulate levels of dopamine in the brain. Thus, the present results should be helpful in optimizing the therapeutic effects of AAV2-hAADC gene therapy, in the context of better management of circulating L-DOPA concentration. And finally, we realize that despite the usefulness of rotational performance to L-DOPA in the present paradigm, the use only one behavioral test is not enough to fully characterize the anti-parkinsonian benefits of AAV2-hAADC gene therapy. Thus, behavioral tests, to further assess motor functions and evaluate possible development of L-DOPA-induced dyskinesia will be taken into account in our future animal studies.

## Supporting Information

S1 Fig Behavioral effect of the repeated carbidopa withdrawal. Pilot study protocol: Animals (n = 6) received an injection of 6-OHDA in left striatum (10 μg). Four weeks after intoxication, severe dopamine depletion in nigrostriatal pathway was confirmed by amphetamine test (5 mg/kg i.p.) in 4 rats. Five weeks after intoxication, animals received stereotactic injection of 7 x 10^10^ vector genomes (vg) of AAV2-hAADC into left striatum. Four weeks later, daily i.p. injections of L-DOPA (5 mg/kg) co-administered with carbidopa (1.25 mg/kg) were initiated, and L-DOPA-induced rotational responses were recorded on days 1, 5, 10, 11, 12, 17, 18, and 19. L-DOPA/carbidopa-treated rats showed rapid sensitization of rotational responses. On days 11 and 18, carbidopa was withdrawn from daily L-DOPA regimen (black column in the graph). Repeatedly, the absence of carbidopa in L-DOPA challenge resulted in an almost complete disappearance of circling behavior in AAV2-hAADC-lesioned rats. Significant differences were determined by non-parametric Kruskal Wallis analysis of variance at each test day with Mann-Whitney.
(TIF)
