# Supplementary figures and images for: Carbidopa-Based Modulation of the Functional Effect of the AAV2-hAADC Gene Therapy in 6-OHDA Lesioned Rats
Source: PLoS One. 2015 Apr 10;10(4):e0122708. doi: 10.1371/journal.pone.0122708 (PMC4393141; doi:10.1371/journal.pone.0122708)

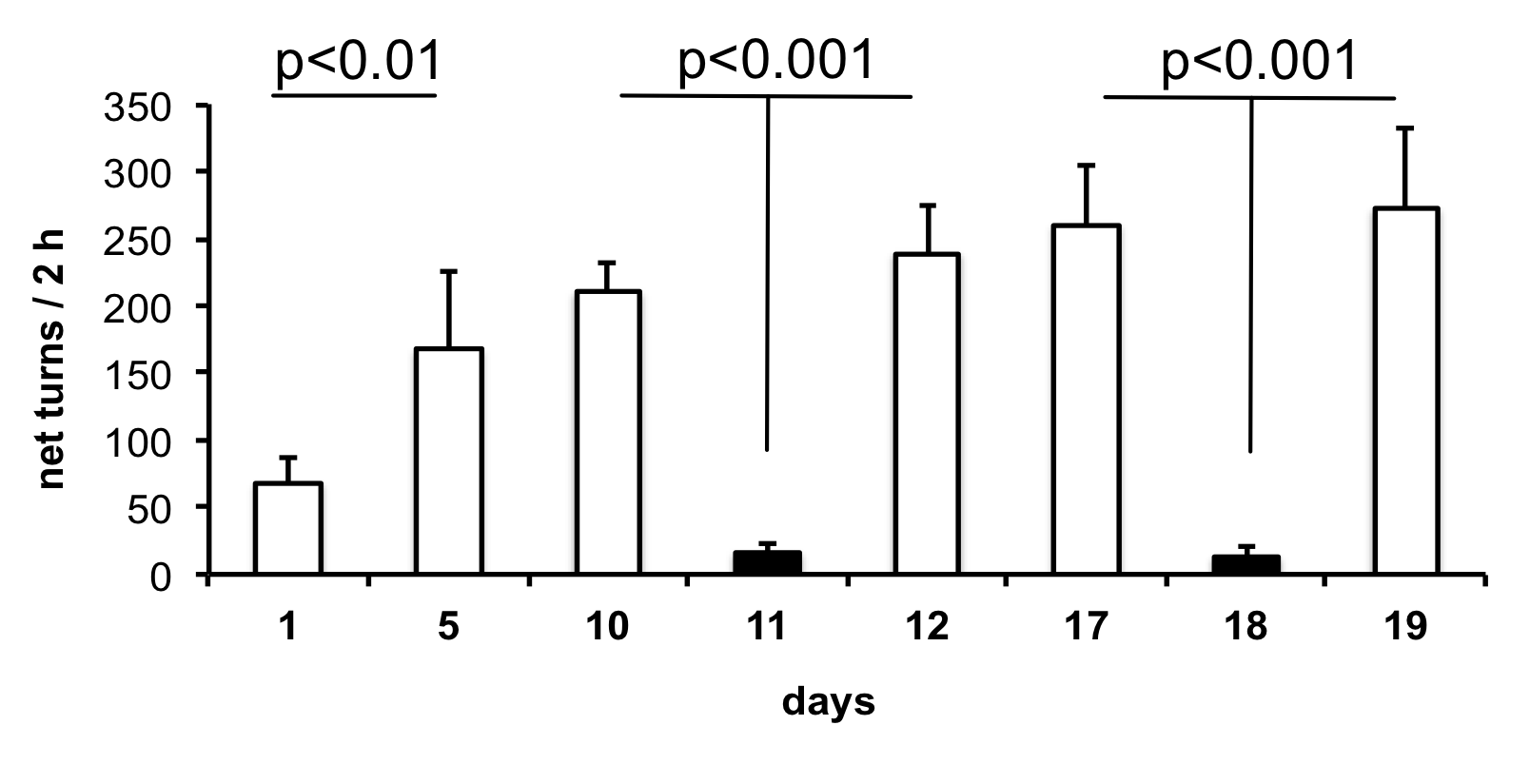

Supplement: S1 Fig — Pilot study protocol: Animals (n = 6) received an injection of 6-OHDA in left striatum (10 μg). Four weeks after intoxication, severe dopamine depletion in nigrostriatal pathway was confirmed by amphetamine test (5 mg/kg i.p.) in 4 rats. Five weeks after intoxication, animals received stereotactic injection of 7 x 10^10^ vector genomes (vg) of AAV2-hAADC into left striatum. Four weeks later, daily i.p. injections of L-DOPA (5 mg/kg) co-administered with carbidopa (1.25 mg/kg) were initiated, and L-DOPA-induced rotational responses were recorded on days 1, 5, 10, 11, 12, 17, 18, and 19. L-DOPA/carbidopa-treated rats showed rapid sensitization of rotational responses. On days 11 and 18, carbidopa was withdrawn from daily L-DOPA regimen (black column in the graph). Repeatedly, the absence of carbidopa in L-DOPA challenge resulted in an almost complete disappearance of circling behavior in AAV2-hAADC-lesioned rats. Significant differences were determined by non-parametric Kruskal Wallis analysis of variance at each test day with Mann-Whitney. (TIF) [file pone.0122708.s001.tif]
